# Tumor Associated Macrophages Protect Colon Cancer Cells from TRAIL-Induced Apoptosis through IL-1β- Dependent Stabilization of Snail in Tumor Cells

**DOI:** 10.1371/journal.pone.0011700

**Published:** 2010-07-22

**Authors:** Pawan Kaler, Vincent Galea, Leonard Augenlicht, Lidija Klampfer

**Affiliations:** Department of Oncology, Albert Einstein Cancer Center, Montefiore Medical Center, New York, New York, United States of America; University of Hong Kong, Hong Kong

## Abstract

**Background:**

We recently reported that colon tumor cells stimulate macrophages to release IL-1β, which in turn inactivates GSK3β and enhances Wnt signaling in colon cancer cells, generating a self-amplifying loop that promotes the growth of tumor cells.

**Principal Findings:**

Here we describe that macrophages protect HCT116 and Hke-3 colon cancer cells from TRAIL-induced apoptosis. Inactivation of IL-1β by neutralizing IL-1β antibody, or silencing of IL-1β in macrophages inhibited their ability to counter TRAIL-induced apoptosis. Accordingly, IL-1β was sufficient to inhibit TRAIL-induced apoptosis. TRAIL-induced collapse of the mitochondrial membrane potential (Δψ) and activation of caspases were prevented by macrophages or by recombinant IL-1β. Pharmacological inhibition of IL-1β release from macrophages by vitamin D_3_, a potent chemopreventive agent for colorectal cancer, restored the ability of TRAIL to induce apoptosis of tumor cells cultured with macrophages. Macrophages and IL-1β failed to inhibit TRAIL-induced apoptosis in HCT116 cells expressing dnIκB, dnAKT or dnTCF4, confirming that they oppose TRAIL-induced cell death through induction of Wnt signaling in tumor cells. We showed that macrophages and IL-1β stabilized Snail in tumor cells in an NF-κB/Wnt dependent manner and that Snail deficient tumor cells were not protected from TRAIL-induced apoptosis by macrophages or by IL-1β, demonstrating a crucial role of Snail in the resistance of tumor cells to TRAIL.

**Significance:**

We have identified a positive feedback loop between tumor cells and macrophages that propagates the growth and promotes the survival of colon cancer cells: tumor cells stimulate macrophages to secrete IL-1β, which in turn, promotes Wnt signaling and stabilizes Snail in tumor cells, conferring resistance to TRAIL. Vitamin D_3_ halts this amplifying loop by interfering with the release of IL-1β from macrophages. Accordingly, vitamin D_3_ sensitizes tumor cells to TRAIL-induced apoptosis, suggesting that the therapeutic efficacy of TRAIL could be augmented by this readily available chemopreventive agent.

## Introduction

Inflammation contributes to tumor progression by establishing conditions that support tumor cell growth and survival and increase their metastatic potential. Indeed, chronic inflammation has been shown to predispose to development of a variety of tumors, a striking example being inflammatory bowel disease, which is associated with elevated risk of colon cancer [1]. Moreover, it appears that colon cancers that do not develop as a complication of inflammatory bowel disease are also driven by inflammation, because it has been shown that regular use of NSAIDs lowers the mortality from sporadic colon cancer and results in regression of adenomas in FAP patients, who inherit a mutation in the Apc gene [2]. Soluble factors which propagate inflammation can be produced by tumor cells themselves or, more often, by cells recruited to the tumor microenvironment, such as tumor associated macrophages (TAMs). Coordinated signaling between tumor cells and nonmalignant cells in the tumor microenvironment is required for the progression of tumors, and signaling pathways that regulate the crosstalk between colon tumor cells and stroma, such as NF-κB and STAT3, have emerged as important targets for chemopreventive and chemotherapeutic agents [3], [4]. Likewise, TNFα antagonists are in phase I/II clinical trials and have been shown to be well tolerated in patients with solid tumors [5], [6].

We recently established that macrophages promote Wnt signaling in colon cancer cells and thus enhance their proliferation, and demonstrated that macrophages exert their protumorigenic activity mainly through the release of IL-1β [7], [8]. Here we show that macrophage-derived factors, in addition to supporting the growth of tumor cells, also promote their survival upon treatment with TNF-related apoptosis inducing ligand (TRAIL), a potent initiator of the extrinsic pathway of apoptosis.

TRAIL initiates apoptosis by binding to two death receptors, DR4 and DR5, while binding to the decoy receptors which lack the death domain, such as DCR1, DCR2 and osteoprotegerin, inhibits its pro-apoptotic activity [9]. Binding of TRAIL to the death inducing receptors DR4/DR5 results in the recruitment of the Fas –associated death domain (FADD) to the receptors, which initiates binding of procaspase-8 and procaspase-9, and the formation of the death inducing signaling complex (DISC) [9]. In type I cells, caspase-8 activation is sufficient to activate effector caspases 3, 6 and 7, while in type II cells, the apoptotic cascade requires integration of the mitochondrial pathway mediated by caspase-8 induced cleavage of Bid.

Tumor cells are significantly more sensitive to TRAIL-induced apoptosis than normal cells, establishing TRAIL and DR4 or DR5 agonistic antibodies as attractive anti-cancer drugs. Indeed, in stark contrast to other members of the TNF family, treatment of mice and primates with recombinant TRAIL induced significant regression of tumors without systemic toxicity [10], [11]. Recently, the combination of TRAIL with all trans-retinyl acetate (RAc) has been shown to induce apoptosis selectively in adenomatous polyposis (APC) deficient epithelial cell without harming normal cellsαα and treatment of Apc*^Min^* mice with TRAIL and RAc induced apoptosis in intestinal polyps and prolonged animal survival [12].

However, there are significant differences in TRAIL sensitivity among human cancer cells. Resistance to TRAIL has been shown to develop in cells with mutant DR5 [13] or in mismatch repair deficient tumors with Bax mutations [14]. In contrast, c-Myc promotes the responsiveness to TRAIL by inhibiting the expression of FLIP, an inhibitor of TRAIL signaling [15], and by counteracting TRAIL-induced upregulation of mcl-1 and cIAP2, two proteins with intrinsic ability to inhibit apoptosis [16]. In addition, stromal cells and soluble factors present in the tumor microenvironment have been shown to have a significant impact on the sensitivity of tumor cells to therapeutic agents [17], [18].

TRAIL deficient mice display increased susceptibility to carcinogen induced tumorigenesis and have increased metastatic potential [19], [20], demonstrating that TRAIL exerts tumor suppressor activity, and suggests an important role of endogenous TRAIL in tumor surveillance. Indeed, the authors showed that TRAIL is, at least in part, responsible for NK cell mediated, IFNγ dependent, mechanism of tumor elimination.

In this study we demonstrated that TRAIL induced apoptosis of colon cancer cells is inhibited by macrophage derived IL-1β, and showed that macrophages and recombinant IL-1β counteract TRAIL-induced apoptosis through activation of Wnt signaling and stabilization of Snail in tumor cells. Finally, we present data indicating that “normalization” of the tumor microenvironment by vitamin D_3_, a potent chemopreventive agent, restores the sensitivity of colon cancer cells to TRAIL, suggesting that the therapeutic efficiency of TRAIL may be greatly improved by agents that inhibit the crosstalk between tumor cells and the tumor microenvironment.

## Materials and Methods

### Cell lines and co-culture experiments

The HCT116 and Hke-3 colorectal carcinoma cell lines, which differ only by the presence of the mutant k-Ras allele [21] and SW480 cells were cultured in MEM, while 293T cells were cultured in DMEM. The human monocytic cell line, THP1, was cultured in RPMI. Normal human monocytes, >90% CD14 and CD11c positive and less than 1% anti T cell receptor positive, were purchased from *Astarte Biologics* (Redmond, WA). Tumor cells and monocytes/macrophages were co-cultured separated by transwell inserts of a polycarbonate membrane with 0.4 µM pore size, which preclude direct cell-cell contact, but permit the exchange of soluble factors (Corning Incorporated, Lowell, MA).

For clonogenic assay, HCT116 and Hke-3 cells were seeded at a density of 200 cells per well of a six well plate alone or together with THP1 macrophages or peripheral blood monocytes for 7 days. Tumor cells were cultured with THP1 monocytes directly (1600 per well of a 6 well plate), as THP1 cells did not attach and form colonies. The optimal ratio between tumor cells and macrophages was established previously [7], [8]. Colonies were fixed and stained with 6% glutaraldehyde and 0.5% crystal violet and counted using Total Lab 1.1 software (Nonlinear Dynamics, Durham, NC, USA).

### Apoptosis assay

Cells were treated with recombinant TRAIL (50 ng/ml, which we determined was the optimal concentration) alone or in the presence of macrophages, IL-1β (5 ng/ml) or TNFα (10 ng/ml) for 7 hours. Cells were resuspended in hypotonic buffer (0.1% Triton X-100, 0.1% sodium citrate) and stained with propidium iodide (50 µg/ml) for 4 hours at 4°C as described [22]. Samples were analyzed by flow cytometry and cell cycle distribution and the extent of apoptosis (cells with a subG1 DNA content) were analyzed by the *Modfit* software. Mitochondrial membrane potential was determined by flow cytometry using the fluorescent dye JC-1 (*Invitrogen*). Cells were stained with 1 µM of JC1 for 1 hour at 37°C, washed with PBS and analyzed by fluorescence in the FL2 channel. Cells were treated with TRAIL for ∼7 hours and were collected for evaluation based on morphological criteria. However, as PI staining may underestimate the amount of apoptosis [23], we confirmed the apoptotic nature of cells by biochemical analysis of cell lysates. Statistical analysis was performed using unpaired Student's t test, with values <0.05 considered statistically significant.

### Transient transfection and Reporter gene assay

HCT116 and Hke-3 cells were transiently transfected with the TOP-FLASH or TOP-FOP luciferase reporter plasmids using the calcium phosphate method. Transfection efficiency was normalized by co-transfection with pTK-Renilla and luciferase activity was determined according to the vendor's protocol (Dual Luciferase reporter assay, Promega, Madison, WI). Dominant negative IκBα was expressed from a plasmid coding for IκBα with serines 32 and 36 mutated to alanine, which confers resistance to stimulus induced degradation [24]. Plasmids expressing constitutively active AKT, (HA-mdelta (4-129) PH-AKT), and dominant negative AKT (HA-AKT-K179M) were provided by Richard Roth [25], [26]. dnTCF4 was described previously [27].

Macrophages were transfected with 20 nM of non specific siRNA (NSP) or siRNAs specific for VDR, IL-1β or STAT1 (Dharmacon, Lafayette, CO) using Lipofectamin LTX (Invitrogen, Carlsbad, CA). The efficiency of silencing was monitored by immunoblotting (for STAT1 and VDR), or by ELISA (for IL-1β), as reported [7], [8].

### Immunofluorescence

For the subcellular detection of β-catenin by immunofluorescence, cells were fixed in 4% paraformaldehyde for 30 minutes. The cells were incubated with anti-β-catenin antibody (1∶100) for 1 h at 37°C and with secondary anti-rabbit antibody conjugated to FITC for 45 min at 37°C. Images were acquired with a SPOT CCD camera and analyzed by SPOT software.

### Western Blot

Western blots were performed using standard procedures. Membranes were blocked with 5% milk in TBS containing 0.1% Tween 20, and incubated with antibodies specific for caspase 8 (Abcam), caspase 9, PARP, Snail (Cell Signaling Technology), pGSK3β (Millipore, Billerica, MA), total β-catenin (BD Biosciences, San Jose, CA), and β-actin (Sigma Aldrich, St. Louis, MO). Immunoreactive bands were visualized by chemiluminescence (Amersham ECLTM western blotting detection kit, Piscataway, NJ).

## Results

### Macrophages and IL-1β protect colon cancer cells from TRAIL induced apoptosis

**Figure 1:**
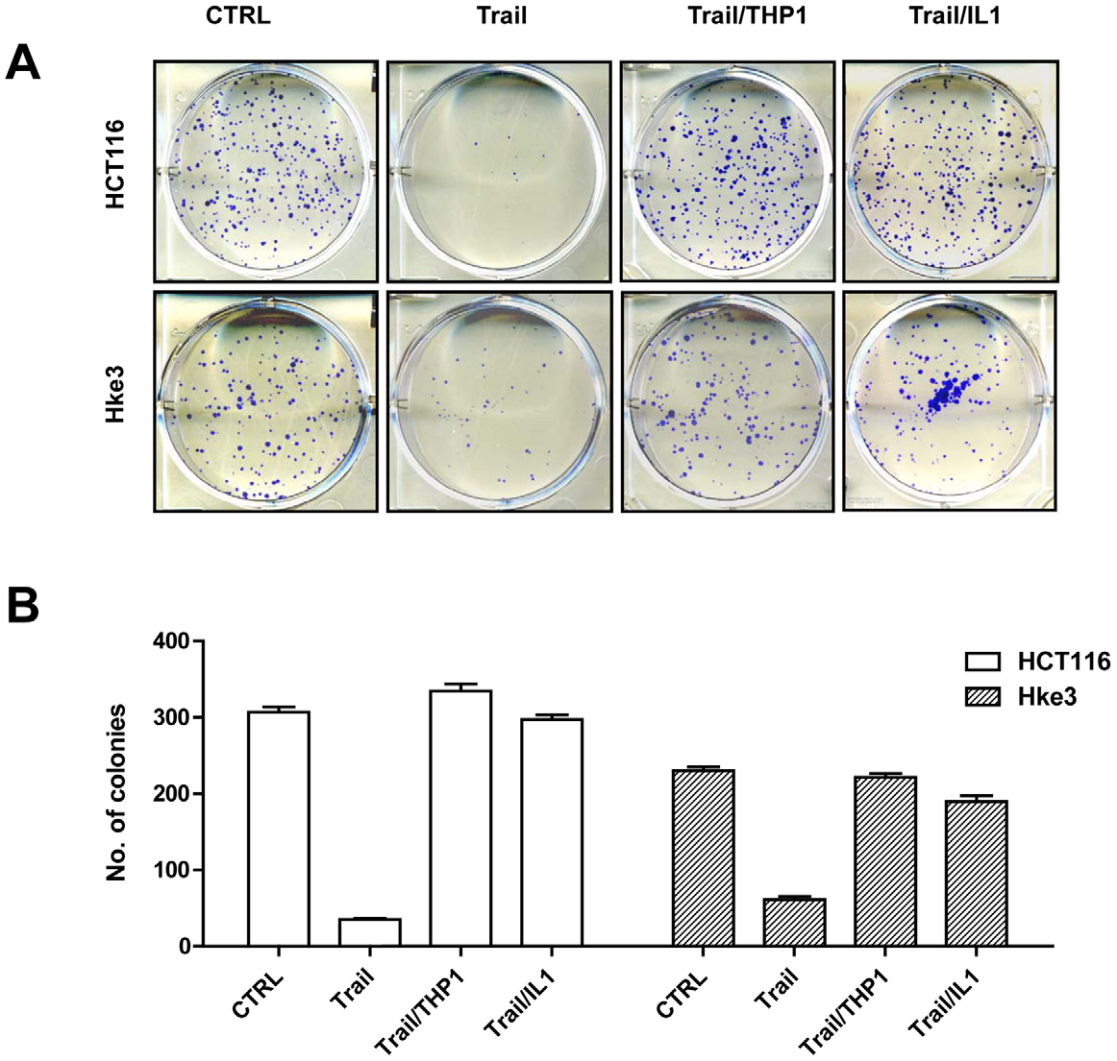
Macrophages and IL-1β counteract TRAIL-induced inhibition of colon cancer growth. A and B: HCT116 and Hke-3 cells were seeded at a density of 200 cells per well of a six well plate in the absence or the presence of macrophages (1600/well) or IL-1β, and were treated with TRAIL for 4 hours. The results shown in B represent the average of two independent experiments each performed in duplicate.

**Figure 2:**
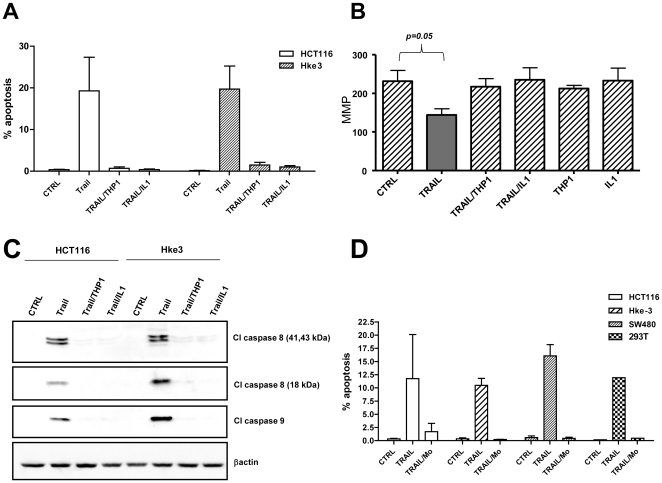
Macrophages and macrophage-derived factors protect colon cancer cells from TRAIL-induced apoptosis. A: HCT116 (average of 5 independent experiments) and Hke-3 cells (average of 4 independent experiments) were treated with TRAIL in the absence or the presence of macrophages or IL-1β (5 ng/ml) as indicated and the extent of apoptosis was determined after 7 hours. B: The mitochondrial membrane potential (MMP) was determined by JC1 staining 5 hours after treatment. C: Activation of caspase 8 and caspase-9 was determined in colon cancer cells treated with TRAIL (50 ng/ml) under conditions indicated. D: TRAIL- sensitive cancer cell lines were treated with TRAIL in the absence or the presence of human peripheral blood monocytes (Mo) and the extent of apoptosis was determined 7 hours after treatment. Data shown represent the average of 2 or 3 independent experiments.

We demonstrated that macrophages induce several prosurvival signaling pathways in colon cancer cells, including NF-κB, AKT and Wnt signaling [7], [8], suggesting that the presence of macrophages could affect the response of tumor cells to therapeutic agents. HCT116 colon cancer cells are highly susceptible to TRAIL-induced apoptosis [16]. Accordingly, TRAIL significantly inhibited the clonogenic growth of HCT116 and HKe-3 cells (Fig. 1A and B), cell lines that differ only by the presence of the mutant kRas [21]. There was no reproducible difference between the responsiveness of HCT116 and Hke-3 cells to TRAIL (Fig. 1 and 2), demonstrating that the presence of mutant kRas does not regulate the responsiveness of cells to TRAIL. To determine whether macrophages alter the sensitivity of tumor cells to TRAIL, we treated HCT116 and Hke-3 cells with TRAIL in the absence or in the presence of THP1 macrophages. Remarkably, the presence of THP1 macrophages or treatment with IL-1β, a cytokine produced in cocultures of tumor cells and macrophages [7], restored the clonogenic growth of TRAIL-treated colon cancer cells (Fig. 1).

Macrophages did not inhibit the expression of DR4 or DR5, or modulate the levels of decoy receptors DcR1 or DcR2 in HCT116/Hke-3 cells (Figure S1), suggesting that they do not modulate the responsiveness to TRAIL through regulation of TRAIL receptors.

Next we showed that macrophages and IL-1β inhibited TRAIL-induced apoptosis of HCT116 and Hke-3 cells (Fig. 2A). THP1 macrophages and IL-1β precluded TRAIL-induced collapse of the mitochondrial membrane potential (MMP) (Fig. 2B) and prevented TRAIL-induced activation of caspase-8 and caspase-9 (Fig. 2C). The human monocytic cell line THP-1 was derived from the peripheral blood of a patient with acute monocytic leukemia and THP1 cells have properties of human monocyte-derived macrophages [28]. These cells have several characteristics of tumor associated macrophages (TAMs), including impaired activation of NF-κB, lack of nitric oxide production in response to LPS/IFNγ (not shown) and high constitutive STAT1 signaling [7], [8]. However, to confirm that the results obtained using THP1 cells are biologically relevant, we showed that normal peripheral blood monocytes, precursors of the tumor associated macrophages, protected a panel of colon cancer cell lines from TRAIL-induced apoptosis (Fig. 2D). Like THP1 macrophages, normal peripheral blood monocytes restored the MMP and prevented activation of caspase 8 and cleavage of PARP in TRAIL-treated tumor cells (data not shown, Figure S2), which is consistent with the ability of colon cancer cells to induce IL-1β release from peripheral blood monocytes [7].

Peripheral blood monocytes inhibited TRAIL-induced apoptosis also in 293T cells (Fig. 2D), demonstrating that macrophage-derived factors are general and potent inhibitors of TRAIL-induced cell death.

### Macrophages protect from TRAIL-induced apoptosis through IL-1β

**Figure 3:**
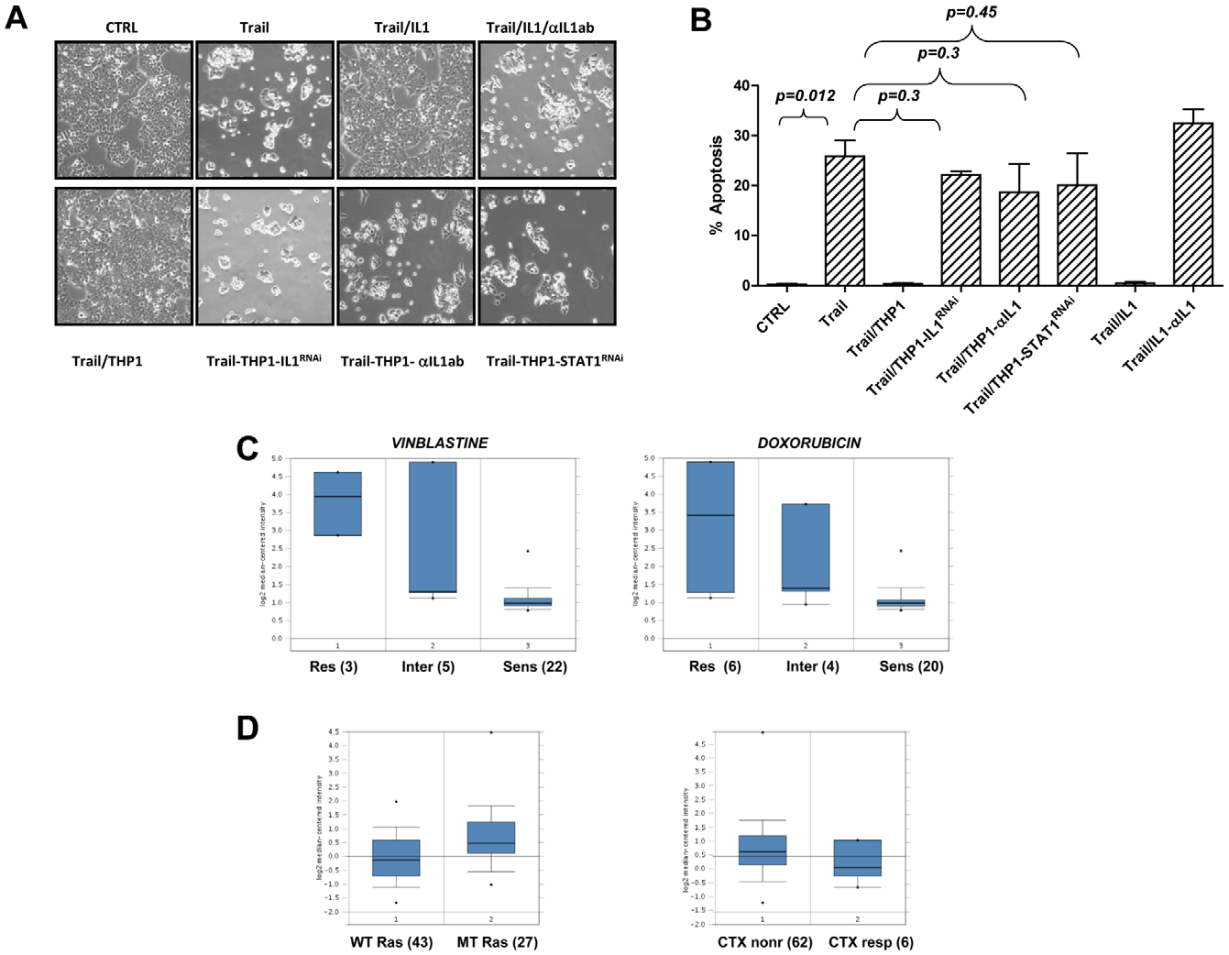
IL-1β is sufficient and required to protect cells from TRAIL-induced apoptosis. A and B: HCT116 cells were cultured with THP1 macrophages with silenced IL-1β or STAT1 expression and were treated with TRAIL as indicated. IL-1β was neutralized by anti-IL-1β specific antibody. Pictures were taken (A) and the amount of apoptosis (B) was determined after 7 hours. Data shown in B represent the average of three independent experiments. C and D: Studies by Gyorffy *et al* ([29], C) and by Khambata-Ford *et al* ([30], D) were surveyed through Oncomine and indicate that cell lines (C) and primary colon cancers (D) with higher levels of IL-1βdisplay resistance to therapeutic agents, such as vinblastine, doxorubicin and cetuximab (CTX).

We showed that IL-1β is sufficient to inhibit TRAIL-induced apoptosis (Fig. 2). To establish whether IL-1β is required for macrophage mediated protection of tumor cells from TRAIL-induced apoptosis, we first silenced IL-1β in THP1 macrophages. These experiments revealed that IL-1β deficient macrophages fail to protect tumor cells from TRAIL-induced apoptosis (Fig. 3A and 3B). Consistent with these data, neutralization of IL-1β by IL-1β specific antibody, or silencing of STAT1, a transcription factor that we showed is required for the release of IL-1β from macrophages [7] also inhibited the anti-apoptotic activity of macrophages (Fig. 3A and 3B). As expected, neutralizing IL-1β antibody inhibited the prosurvival activity of IL-1β, demonstrating the specificity of antibody. Together, these data established that tumor associated macrophages protect tumor cells from TRAIL-induced apoptosis in an IL-1β dependent manner.

To determine whether the ability of IL-1β to interfere with therapy induced apoptosis is restricted to TRAIL, we surveyed Oncomine (www.oncomine.org), which is an assembly of human tumor microarray studies. The analysis of 30 cell lines by Gyorffy *et al* [29] revealed that high expression of IL-1β in tumor cells lines strongly correlates with the resistance of cells to vinblastine and doxorubicin (Fig. 3C). The analysis of 70 metastatic colon tumors [30] revealed that colon tumors that harbor mutant kRas have higher levels of IL-1β than tumors with WT kRas (Fig. 3D, left panel). Whether IL-1β is produced by stromal cells or tumor cells themselves cannot be concluded, however the analysis of this database revealed that higher levels of IL-1β in tumors correlate with resistance to cetuximab [30] (Fig. 3D, right panel). In summary, these data established that the levels of IL-1β are elevated in a subset of human colon tumors, and that IL-1β modulates the response of tumor cells to a variety of therapeutic agents both *in vitro* and *in vivo*.

### Pharmacological inhibition of IL-1β stimulation by Vitamin D_3_ inhibits the prosurvival activity of macrophages

**Figure 4:**
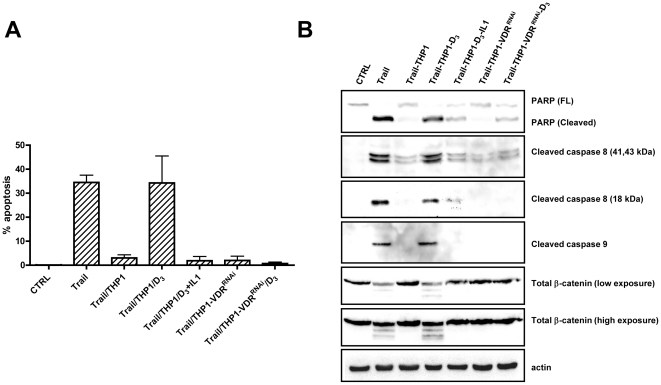
Pharmacological inhibition of IL-1β secretion by Vitamin D_3_ inhibits the prosurvival function of macrophages. A: The amount of apoptosis was determined in HCT116 cells treated with TRAIL under indicated conditions. Error bars were derived from 2 independent experiments. B: The effect of vitamin D_3_ on TRAIL-induced activation of caspases and the cleavage of PARP and β-catenin was determined by immunoblotting.

We recently demonstrated that the cross talk between tumor cells and macrophages is disrupted by vitamin D_3_, an important chemopreventive agent for colorectal cancer. Vitamin D_3_ inhibited the ability of tumor cells to stimulate IL-1β release from macrophages [7] and, subsequently, vitamin D_3_ treated macrophages failed to induce Wnt signaling in tumor cells [7]. These data suggested that vitamin D_3_ may also regulate TRAIL induced apoptosis. HCT116 cells were cultured alone or in the presence of macrophages, and were treated with TRAIL in the absence or the presence of vitamin D_3_. Vitamin D_3_ did not affect TRAIL-induced apoptosis in HCT116 or Hke-3 cells (data not shown), which is consistent with the unresponsiveness of these cells to vitamin D_3_ [31]. However, we demonstrated that the ability of macrophages to protect colon cancer cells from TRAIL-induced apoptosis was inhibited by vitamin D_3_ (Fig. 4A). The addition of exogenous IL-1β prevented the ability of vitamin D_3_ to regulate TRAIL induced apoptosis, confirming that vitamin D_3_ restored the sensitivity of tumor cells to TRAIL by inhibiting the release of IL-1β from macrophages, and not by affecting signaling by IL-1β. Silencing of VDR by VDR specific siRNA in macrophages did not affect their ability to inhibit TRAIL-induced apoptosis in tumor cells, however vitamin D_3_ failed to restore TRAIL induced apoptosis of tumor cells cocultured with VDR deficient macrophages (Fig. 4A). These data demonstrated that vitamin D_3_ affects the crosstalk between tumor cells and macrophages by targeting macrophages and not the tumor cells, and that, accordingly, it requires the expression of VDR on macrophages, consistent with our published data [7].

Biochemical analysis confirmed that while macrophages inhibited TRAIL-induced activation of caspase 8 and caspase-9 and cleavage of PARP and β-catenin, treatment with vitamin D_3_ promoted TRAIL-mediated activation of the apoptotic cascade in tumor cells that were grown in the presence of WT, but not VDR deficient macrophages (Fig. 4B). As vitamin D_3_ acts as an inhibitor of IL1-β release, its activity was prevented by exogenous IL1β and was dependent on the expression of VDR on macrophages (Fig. 4A, Fig. 4B) and not on tumor cells (data not shown).

Vitamin D_3_ also restored sensitivity to TRAIL in colon cancer cells that were cultured in the presence of peripheral blood monocytes. It enhanced activation of caspase 8 and subsequent cleavage of PARP and β-catenin in HCT116 that acquired resistance to TRAIL due to the presence of peripheral blood monocytes (Figure S2).

These data suggest that the therapeutic efficacy of TRAIL could be greatly improved by vitamin D_3_ in cancers that are infiltrated by macrophages.

### Wnt signaling is required for the prosurvival activity of macrophages

**Figure 5:**
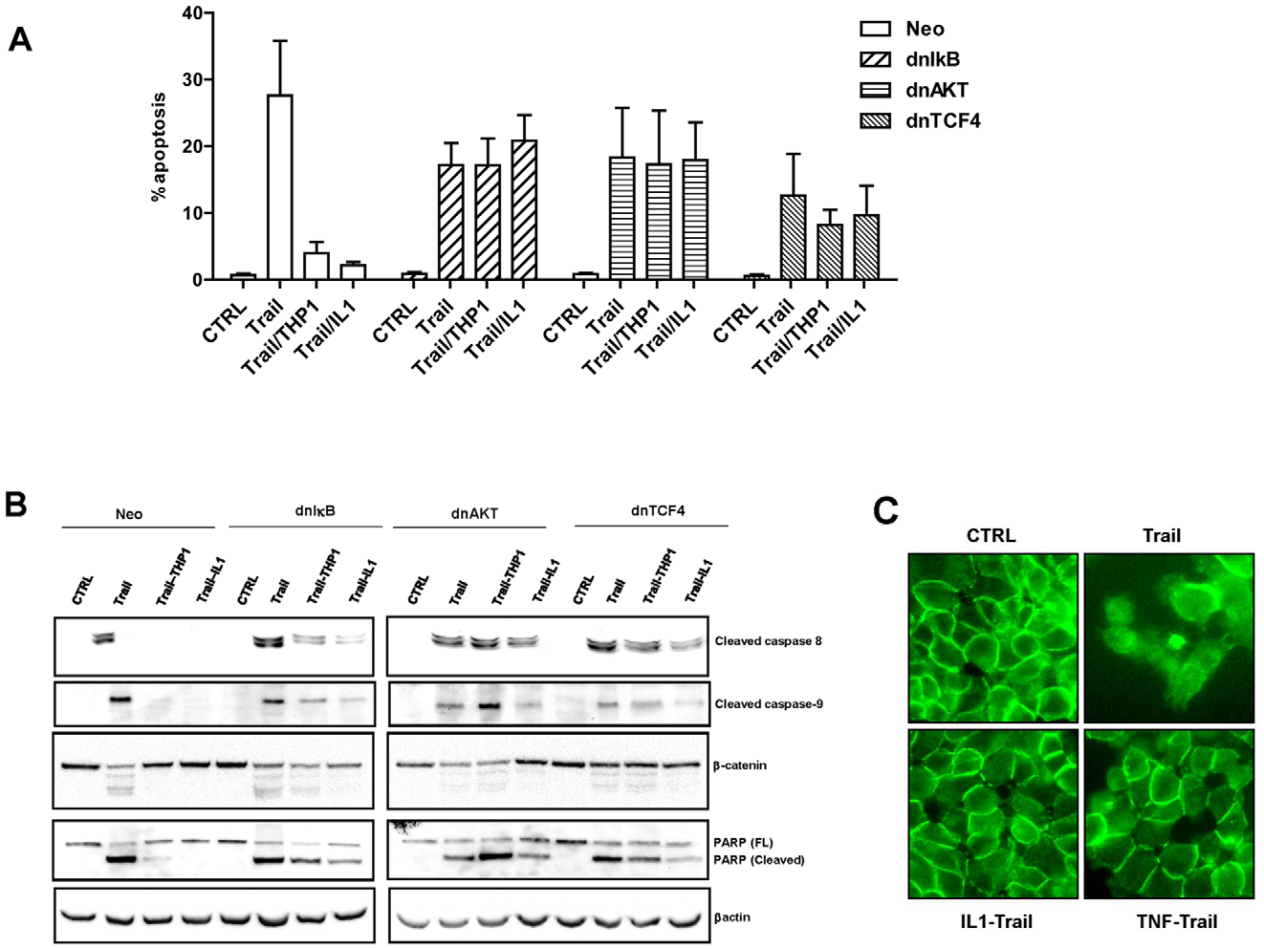
Wnt is required for the prosurvival activity of macrophages and IL-1β. A and B: HCT116 expressing dnIκB, dnAKT or dnTCF4 were treated with TRAIL under indicated conditions and the amount of apoptosis was determined by propidium iodide staining. A: Data represent the average of 5 independent experiments. B: The extent of caspase activation and the cleavage of PARP and β-catenin were determined in cell lysates by immunoblotting. C: The localization of β-catenin was determined by immunofluorescence in HCT116 cells treated with TRAIL in the absence or the presence of IL-1β or TNFα.

We recently demonstrated that macrophages and IL-1β induce Wnt signaling in colon cancer cells through activation of NF-κB-dependent AKT signaling [8], pathways that have been shown to protect tumor cells from apoptosis. To determine whether macrophages and IL-1β inhibit TRAIL-induced apoptosis through activation of these pro-survival signaling pathways, we expressed in HCT116 cells dnIκB (an inhibitor of NF-κB signaling), dnAKT (an inhibitor of AKT signaling) and dnTCF4 (an inhibitor of Wnt signaling). Inhibition of NF-κB or AKT did not impact TRAIL-induced apoptosis. However, cells transfected with dnTCF4 reproducibly showed a modestly reduced amount of TRAIL-induced apoptosis (differences were not statistical significant), which may be consistent with the requirement of the Wnt pathway for optimal TRAIL-induced apoptosis [32] (Fig. 5A). However, the critical point was that in contrast to cells transfected with an empty plasmid, macrophages and IL-1β did not protect cells with impaired NF-κB, AKT or Wnt signaling from TRAIL-induced apoptosis (Fig. 5A). We confirmed that macrophages and IL-1β failed to counteract TRAIL-induced activation of caspase-8 and caspase-9 and subsequent cleavage of PARP in cells that express either dnIκB, dnAKT or dnTCF4 (Fig. 5B). Of particular interest, macrophages and IL-1β prevented TRAIL-induced cleavage of β-catenin, but only in cells with intact NF-κB/AKT/Wnt signaling (Fig. 5B).

Cleavage of β-catenin was mediated by caspases, as we could prevent its processing by ZVAD, a pan-caspase inhibitor (Figure S3). Cleaved β-catenin has been shown to exhibit impaired transcriptional activity [33]. We showed that TRAIL inhibits TCF4/β-catenin transcriptional activity and that macrophages and IL-1β could partially restore β-catenin transcriptional activity in TRAIL-treated cells (Figure S3).

Similar to published reports [34] we found that, despite the fact that HCT116 cells carry a mutant β-catenin allele, most of β-catenin is localized to the membranes in HCT116 cells. Consistent with the cleavage of β-catenin by TRAIL, membranous localization of β-catenin was severely diminished in TRAIL treated cells (Fig. 5C). Both IL-1β and TNFα restored the membranous localization of β-catenin in HCT116 cells in TRAIL-treated cells (Fig. 5C), in accordance with their prosurvival activity.

Together, these data demonstrated that tumor associated macrophages (TAMs) protect tumor cells from TRAIL-induced apoptosis through their ability to stabilize β-catenin and to promote Wnt signaling in colon cancer cells.

### Macrophages, TNFα and IL-1β stabilize Snail in an NF-κB and AKT/WNT dependent manner

**Figure 6:**
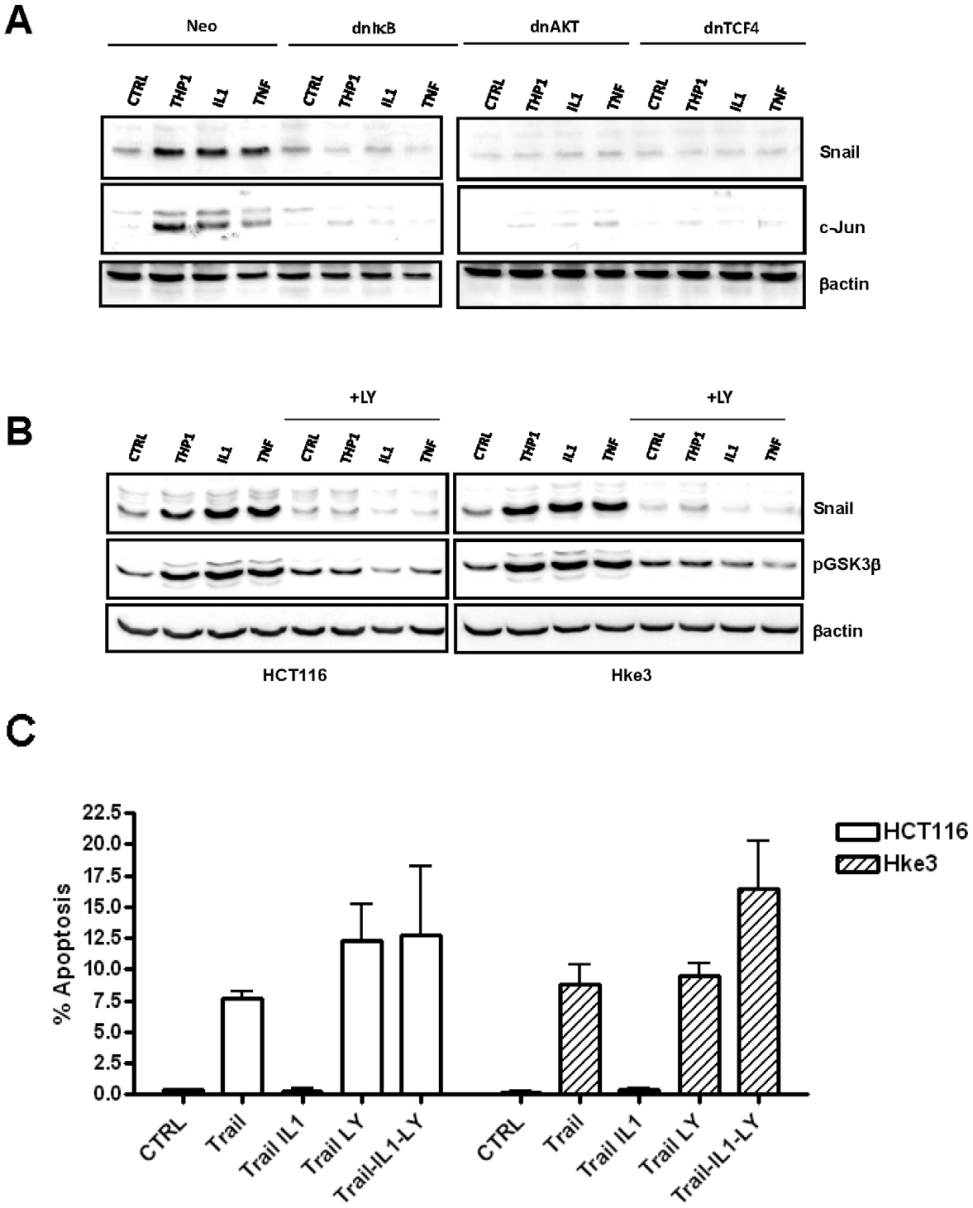
Macrophages, IL-1β and TNFα stabilize Snail in a NF-κB/AKT/Wnt dependent manner. **A:** Macrophages, IL-1β and TNFα stabilize Snail in a NF-κB/AKT/Wnt dependent manner. HCT116 cells were transfected with dnIκB, dnAKT or dnTCF4 and were treated with IL-1β or TNFα, or were cultured with THP1 macrophages as indicated. The levels of Snail and c-jun were determined by immunoblotting. **B:** The levels of Snail and pGSK3β were determined by immunoblotting in Hke-3 cells that were treated with IL-1β, TNFα or were co-cultured with macrophages in the absence or the presence of LY 294002 for 24 hours. **C**: HCT116 and Hke-3 cells were treated with TRAIL in the absence or the presence of IL-1β and LY294002, as indicated. The experiment was performed three times.

Although macrophages and IL-1β protected tumor cells from TRAIL-induced apoptosis, they did not regulate the levels of the bcl-2 family members in tumor cells, such as Bcl-x or Mcl1, or alter the expression of cIAPs, which have been shown to modulate the response of cells to TRAIL-induced apoptosis [16] (data not shown). However, we showed that the levels of Snail protein were increased in HCT116 cells treated with IL-1β or in HCT116 cells cultured in the presence of macrophages (Fig. 6A). TNFα, another macrophage-derived factor that can induce Wnt signaling in tumor cells [7], [35], also elevated the levels of Snail in tumor cells (Fig. 6A). We demonstrated that macrophages, IL-1β and TNFα elevated Snail through NF-κB, AKT and Wnt signaling, as they failed to increase the levels of Snail in cells expressing dnIκB, dnAKT or dnTCF4 (Fig. 6A). The levels of Snail mRNA in HCT116 or Hke-3 cells were not increased by macrophages (not shown), suggesting that macrophage-derived factors stabilize Snail protein in tumor cells. The induction of c-jun by IL-1β and TNFα also required NF-κB, consistent with published data (Fig. 6A). We showed that TNFα and IL-1β also failed to induce c-jun in cells expressing dnTCF4, confirming the importance of Wnt signaling for the biological activity of these cytokines and for the interaction of tumor cells with macrophages.

GSK3β is a major kinase that phosphorylates Snail and induces its degradation [36], [37]. Because we reported that macrophages and IL-1β inactivate GSK3β in tumor cells [7], [8], we next examined whether macrophages/IL-1β stabilize Snail through inhibition of GSK3β. HCT116 and Hke-3 cells were treated with LY294002, a PI3K inhibitor - and thus activator of GSK3β (phoshorylation of GSK3β on Serine 9 inhibits its activity). As shown in Fig. 6B, treatment of cells with LY294002 indeed precluded macrophages/IL-1β/TNFα-mediated inactivation of GSK3β, and completely prevented stabilization of Snail by macrophages, TNFα and IL-1β. This result demonstrates that inhibition of GSK3β regulates the stability of Snail in HCT116 cells and strongly suggests that macrophages, TNFα and IL-1β stabilize Snail through their ability to inhibit GSK3β. Indeed, pharmacological inhibition of GSK3β by LiCL or by GSK3β specific inhibitor, AR-A014418, was sufficient to stabilize Snail in tumor cells (Figure S4).

Finally, treatment of cells with LY29004 completely prevented the ability of IL-1β to protect colon cancer cells from TRAIL-induced apoptosis (Fig. 6C), implying that macrophages and IL-1β protect from TRAIL induced apoptosis through GSK3β dependent stabilization of Snail.

### Macrophages and IL-1β protect from TRAIL-induced apoptosis through stabilization of Snail

**Figure 7:**
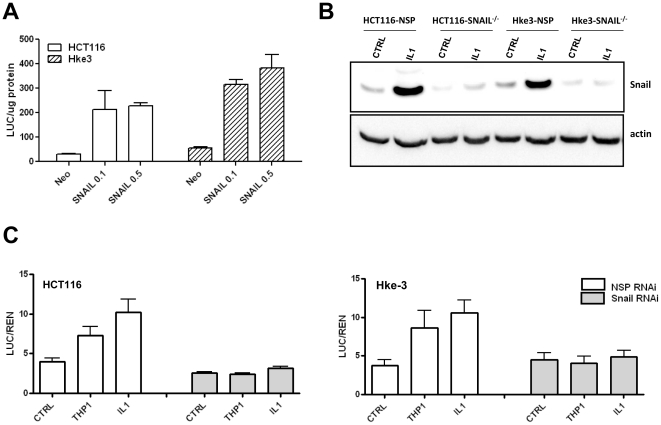
Snail regulates Wnt signaling. A: HCT116 and Hke-3 cells were transfected with TOP-FLASH reporter together with an empty vector (neo), or the increasing concentrations of Snail expression vector. The experiment was performed in triplicate. B: Tumor cells were transfected with NSP or Snail specific siRNA and were treated with IL-1β as indicated. The levels of Snail were determined by immunoblotting. C: Tumor cells were transfected with TOP-FLASH reporter together with NSP or Snail specific siRNA. Data represent the average of two independent experiments each performed in duplicate.

Snail has been shown to interact with β-catenin and to promote the expression of Wnt target genes [38]. In accord with these data, we found that overexpression of Snail promotes TOP-FLASH-driven activity in both HCT116 and Hke-3 cells (Fig. 7A). In order to determine whether stabilization of Snail by macrophages/IL-1β contributes to their ability to drive Wnt signaling, we silenced Snail in tumor cells (Fig. 7B), and examined the ability of macrophages and IL-1β to induce Wnt signaling in Snail deficient HCT116 and Hke-3 cells. In contrast to HCT116 and Hke-3 cells transfected with NSP(nontargeting) RNAi, cells transfected with Snail specific RNAi failed to respond to macrophages and IL-1β with enhanced Wnt signaling (Fig. 7C). Snail deficiency did not impair IL-1β induced NF-κB activation in HCT116 cells (data not shown), demonstrating a specific requirement for Snail in macrophage mediated β-catenin driven transcription.

**Figure 8:**
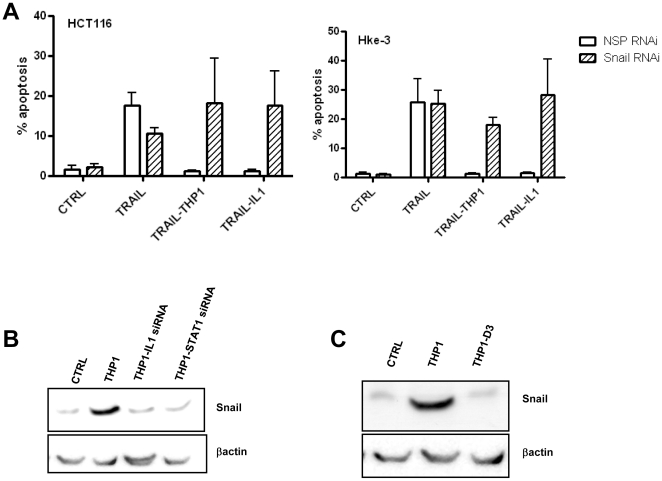
Snail mediates the anti-apoptotic activity of macrophages and IL-1β. A: HCT116 and Hke-3 cells were transfected with NSP or Snail specific siRNA and were treated with TRAIL in the absence or the presence of macrophages or IL-1β as indicated. Data represent the average of two independent experiments. B and C: Silencing of IL-1β or STAT1 (B) in macrophages, or treatment with vitamin D_3_ (C) abrogates the ability of macrophages to stabilize Snail in colon cancer cells.

Finally, since we demonstrated that macrophages and IL-1β protect from TRAIL-induced apoptosis through Wnt signaling (Fig. 5), we tested whether macrophages can protect tumor cells transfected with Snail siRNA from TRAIL-induced apoptosis. Indeed, macrophages and IL-1β failed to inhibit TRAIL induced apoptosis in Snail deficient HCT116 or Hke-3 cells (Fig. 8A), confirming that macrophages and IL-1β protect from TRAIL-induced apoptosis through stabilization of Snail in tumor cells. Indeed, macrophages with silenced IL-1β or STAT1 (Fig. 8B) or vitamin D_3_ treated macrophages (Fig. 8C), which failed to protect tumor cells from TRAIL-induced apoptosis ([7], Fig. 4), also failed to stabilize Snail in tumor cells.

**Figure 9:**
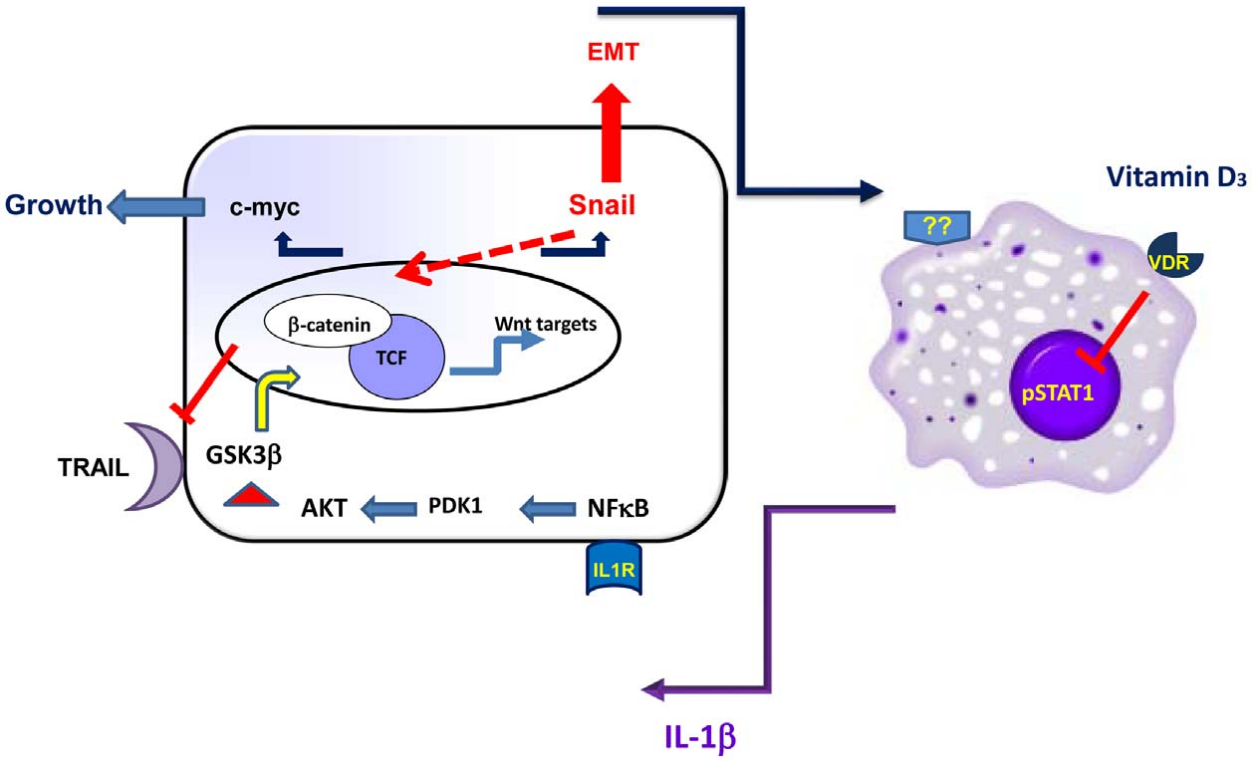
Crosstalk between tumor cells and macrophages which propagates the growth and survival of cancer cells. Tumor cells activate macrophages to secrete IL-1β which in a NF-κB/AKT dependent manner inactivates GSK3β and thus promotes Wnt signaling in tumor cells. Elevated levels of c-myc contribute to enhanced growth of tumor cells, while increased levels of Snail contribute to enhanced Wnt signaling, protect cells from TRAIL induced apoptosis, and are likely to contribute to the epithelial mesenchymal transition (EMT) and enhanced metastatic potential of tumor cells.

These data established a pivotal role of Snail in inflammation-induced resistance of tumor cells to TRAIL (Fig. 9).

## Discussion

Acquired resistance to tumor therapy can develop as a result of *de novo* genetic changes that accompany the progression of cancer. In addition, tumors can attain resistance through a pathway that is not intrinsic to tumor cells, but originates in the tumor microenvironment, also called the tumor microenvironment mediated drug resistance (TMMDR). Two types of TMMDR have been described: resistance that is mediated by soluble factors produced by the cells in the tumor microenvironment and resistance that develops as a result of the adhesion of tumor cells to stromal fibroblasts or to components of the extracellular matrix [18]. It is therefore likely that therapeutic approaches that aim to normalize the tumor microenvironment and/or interrupt the crosstalk between the tumor cells and the microenvironment, will not only help to stall the progression of cancer, but also have a potential to promote sensitivity of tumors to chemotherapy or radiotherapy.

Recently we reported that vitamin D_3_, a potent chemopreventive agent for colorectal cancer, inhibits growth of colon cancer cells by targeting the tumor associated macrophages [7]. We showed that vitamin D_3_ inhibited the ability of tumor cells to induce the release of IL-1β from macrophages, and thereby inhibited the subsequent increase in Wnt signaling and growth of colon cancer cells. Here we present data which establish that TRAIL responsive colon cancer cells acquire resistance to TRAIL when cultured in the presence of macrophages, and showed that macrophage-derived IL-1β was required for their anti-apoptotic activity. Consistent with the ability of vitamin D_3_ to inhibit the release of IL-1β from macrophages, vitamin D_3_ restored the sensitivity of tumor cells to TRAIL. Macrophages were not general inhibitors of apoptosis, as we demonstrated that they actually promoted apoptosis of colon cancer cells in response to 5FU and butyrate (Kaler et al, unpublished). Our preliminary results suggest that macrophages promoted 5FU-induced apoptosis in an IL-1β dependent manner, while the enhancement of butyrate induced apoptosis was mediated though an unknown macrophage-derived factor (Kaler et al, unpublished). This underscores the complexity of the interactions between tumor cells and the stroma, and emphasizes the specificity of the interactions between tumor cells and environmentally derived factors, which is likely to contribute to contrasting reports regarding prognostic significance of macrophages in colon cancer [39]. The levels of IL-1β are increased in colon cancer patients harboring mutant kRas and elevated levels of IL-1β correlate with the resistance to cetuximab (Fig. 3D) [30].

Cytokines secreted by the activated tumor stroma modulate tumor growth and regulate their survival and invasiveness through activation of oncogenic signaling pathways in tumor cells, examples being activation of NF-κB by TNFα and IL-1β, and activation of STAT3 by IL-6 [4], [40]. More recently, TNFα [35], Hepatocyte Growth Factor [41], PDGF [42] and FGF19 [43] and IL-1β [7] have been shown to activate Wnt/β-catenin signaling, the oncogenic pathway activated in the majority of colorectal cancers.

Here we present data which demonstrate that macrophages and IL-1β protect tumor cells from TRAIL-induced apoptosis through induction of Wnt signaling in tumor cells, as cells expressing dnTCF4 were not protected from TRAIL-induced apoptosis by macrophages. Likewise, Wnt expressing rat embryonic fibroblasts have been shown to inhibit TRAIL-induced apoptosis of human leukemia cells [44].

Consistent with our observation that macrophages/IL-1β induce Wnt signaling in an NF-κB dependent manner [8], we showed that macrophages and IL-1β failed to inhibit TRAIL-induced apoptosis in tumor cells expressing dnIκB, confirming the oncogenic role of NF-κB signaling. It has been shown that the ability of LPS, an inducer of IL-1β, to promote the growth of CT26 tumors strictly depends on functional NF-κB signaling, and that inhibition of NF-κB converts inflammation-induced tumor growth to TRAIL-mediated tumor regression [45]. Our data suggest that tumor cells with impaired NF-κB/Wnt signaling fail to respond to survival signals from the tumor microenvironment and thus undergo TRAIL-mediated cell death.

Myc has been shown to sensitize colon cancer cells to TRAIL induced apoptosis through repression of MCL1 and cIAP2 in bax deficient HCT116 cells [16]. In contrast, we have not observed modulation of c-myc, MCL-1 or cIAP1 or cIAP2 in our system, using HCT116 cells which are bax proficient. However, we demonstrated that macrophages, TNFα and IL-1β increased the levels of Snail in tumor cells, a known Wnt target gene and regulator of tumor cell apoptosis [46]. Snail is a zinc finger transcription factor that, through transcriptional repression of E cadherin, induces an epithelial mesenchymal transition (EMT) [47], [48]. Although IL-1β was shown to upregulate Snail and to downregulate E-cadherin in head and neck squamous carcinoma cells [49], our initial analysis revealed no major changes in the expression of epithelial or mesenchymal markers in colon cancer cells exposed to macrophage-derived factors. The only mesenchymal marker strongly induced in these cultures was fibronectin (Kaler, not shown). However, more extensive analyses are required to establish whether stabilization of Snail is sufficient to promote an EMT in colon cancer cells.

GSK3β phosphorylates both the NES (nuclear export sequence) and the destruction box in Snail and thereby triggers ubiquitin mediated proteasomal degradation of Snail [37]. In addition, inhibition of GSK3β has been also shown to activate the transcription of Snail [36]. We showed that pharmacological inhibition of GSK3β (Figure S4) or IL-1β mediated inhibition of GSK3β (Fig. 6), were sufficient to elevate the levels of Snail in colon cancer cells. This is in contrast to the recent report that TNFα stabilized Snail through NF-κB-dependent induction of CSN2 in breast cancer cells, which inhibited the association of Snail with GSK3β and thus suppressed its phosphorylation and degradation [50].

Wnt signaling has been shown to promote transcription, protein stability and to regulate nuclear localization of Snail [36], [37]. In turn, Snail interacts with β-catenin and increases the expression of Wnt target genes [38]. Likewise, Gli1, the hedgehog pathway transcription factor, has been shown to promote Wnt signaling through induction of Snail [51]. We showed that macrophage-induced stabilization of Snail contributes to Wnt signaling in colon cancer cells and creates a positive feedback loop initiated, and propagated, by macrophage-derived IL-1β. In the Apc*^Min^* mouse model of intestinal tumorigenesis, inhibition of Snail increased cell death and reduced tumor formation [52] and in human tumors, nuclear β-catenin activity is enhanced at the invasive front, where the expression of E-cadherin, a direct target of Snail, is downregulated [53], suggesting that this positive regulatory signaling is functional *in vivo*.

Macrophages have been shown to promote invasiveness of breast cancer cells by inducing epithelial mesenchymal transition (EMT) via NF-κB dependent stabilization of Snail [50]. Our data suggest that colon cancer cells with stabilized Snail escape TRAIL-induced cell death, a novel mechanism whereby Snail promotes tumor progression.

Many studies have investigated the role of genetic and epigenetic changes in the responsiveness of tumor cells to TRAIL. This report establishes that the proinflammatory tumor microenvironment can render tumor cells resistant to TRAIL. Consistent with our data, TRAIL induced apoptosis is inhibited by IL8 in ovarian cancer cells [54], [55], by IL-1β in keratinocytes [56], [57], and by IL6 in multiple myeloma cells [58]. Neutralization of IL4 significantly enhanced the effectiveness of TRAIL in epithelial tumors [59], confirming that factors derived from the tumor microenvironment can diminish the response of tumor cells to TRAIL. We established that vitamin D_3_, which interrupts the crosstalk between tumor cells and macrophages, restores the sensitivity of colon cancer cells to TRAIL. Because TRAIL is a promising therapeutic agent, these data offer an exciting opportunity to pharmacologically enhance the responsiveness of cancer patients to TRAIL by commonly used chemopreventive agent. Our data suggest that such an approach should be beneficial in patients with tumors that have a high level of infiltration of macrophages and therefore may not respond to TRAIL alone.

## Supporting Information

Figure S1 The expression of TRAIL receptors on tumor cells: The expression of DR4, DR5, DcR1 and DcR2 mRNA in HCT116 and Hke-3 cells that were cultured alone or together with THP1 macrophages, and were either left untreated or were treated with vitamin D3.
(0.19 MB TIF)

Figure S2 Vitamin D inhibits the anti-apoptotic activity of peripheral blood monocytes (Mo). HCT116 and Hke-3 cells were treated with TRAIL in the absence or the presence of peripheral blood monocytes and vitamin D, as indicated. The extent of apoptosis was determined by PI staining (left panel), and the activation of caspase 8 and cleavage of PARP and beta catenin were determined by immunoblotting (right panel).
(0.12 MB TIF)

Figure S3 TRAIL inhibits beta-catenin/TCF4 transcriptional activity. A: HCT116 and Hke-3 cells were treated with TRAIL in the presence of pan-caspase inhibitor, ZVAD, and the cleavage of PARP and beta-catenin was determined by immunoblotting. B: HCT116 and Hke-3 cells were transfected with the TOP-FLASH reporter gene and were treated with TRAIL (10 ng/ml) in the presence of macrophages or IL1 as indicated for 24 hours.
(0.16 MB TIF)

Figure S4 Inhibition of GSK3beta stabilizes Snail in tumor cells. HCT116 cells were treated with LiCl (10 mM) or with AR-A014418 (AR, 50 mM) for 24 hours and the levels of Snail and beta actin were determined by immunoblotting.
(2.24 MB TIF)
