# Expression of Concern: Tumor Associated Macrophages Protect Colon Cancer Cells from TRAIL-Induced Apoptosis through IL-1β- Dependent Stabilization of Snail in Tumor Cells

**DOI:** 10.1371/journal.pone.0263429

**Published:** 2022-01-27

**Authors:** The PLOS ONE Editors

After publication of this article [1], concerns were raised about some of the western blots in Figs 6, 7 and 8.

Specifically:

- The bands in lanes one, two, four, seven and eight in the HCT116 β-actin panel of Fig. 6B appear similar to the bands in lanes one, two, four, seven and eight in the actin panel of Fig. 7B. The other lanes in these panels appear different comparing Fig. 6B versus 7B.
- The bands in lanes two and three of the β-actin panel in Fig. 8C are duplicates of lanes one and two in the β-actin panel of Fig. 8B.

In response to queries about these figures, the corresponding author acknowledged the similarities in some bands in the β-actin loading controls in Figs. 6B and 7B but noted that there are differences and that in their view it is unlikely that the two panels were generated from the same blot. The corresponding author stated that there was a mistake in assembly of Fig. 8, and the β-actin loading control in Fig. 8C is incorrect, but a replacement image of the correct loading control is not available. The corresponding author also stated that the underlying data for the rest of the study are no longer available.

In light of the above concerns, which cannot be fully resolved because of the unavailability of the original image files underlying Figs 6B, 7B and 8B, the *PLOS ONE* Editors issue this Expression of Concern.

## References

[1] Kaler P, Galea V, Augenlicht L, Klampfer L (2010) Tumor Associated Macrophages Protect Colon Cancer Cells from TRAIL-Induced Apoptosis through IL-1β- Dependent Stabilization of Snail in Tumor Cells. PLoS ONE 5(7): e11700. doi: 10.1371/journal.pone.0011700 20661477 PMC2908545

