# Supplementary figures and images for: Tumor Associated Macrophages Protect Colon Cancer Cells from TRAIL-Induced Apoptosis through IL-1β- Dependent Stabilization of Snail in Tumor Cells
Source: PLoS One. 2010 Jul 22;5(7):e11700. doi: 10.1371/journal.pone.0011700 (PMC2908545; doi:10.1371/journal.pone.0011700)

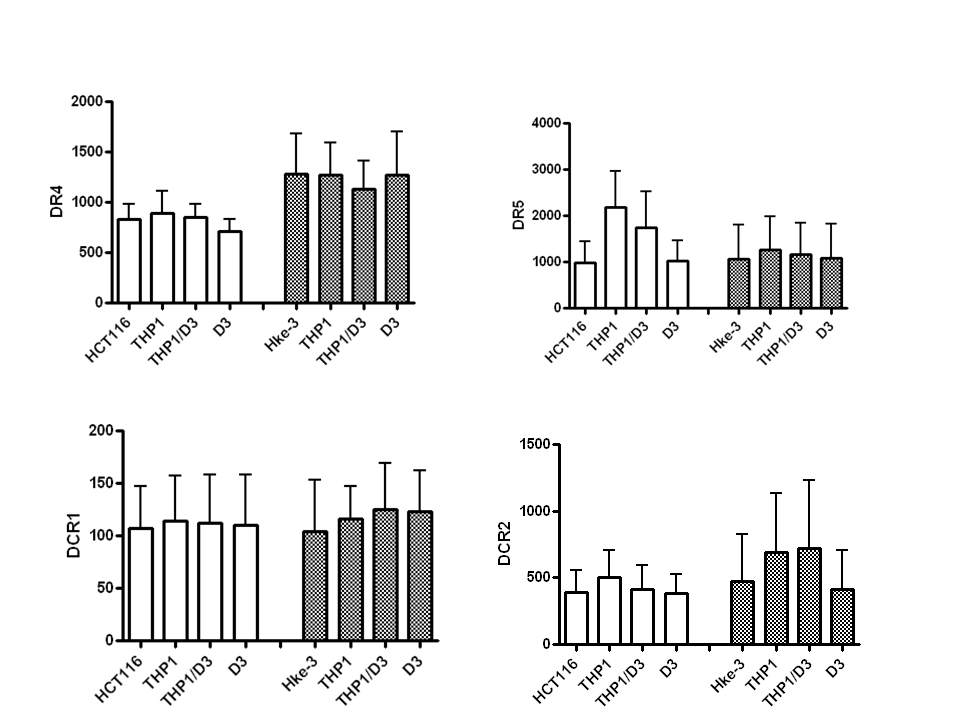

Supplement: Figure S1 — The expression of TRAIL receptors on tumor cells: The expression of DR4, DR5, DcR1 and DcR2 mRNA in HCT116 and Hke-3 cells that were cultured alone or together with THP1 macrophages, and were either left untreated or were treated with vitamin D3. (0.19 MB TIF) [file pone.0011700.s001.tif]

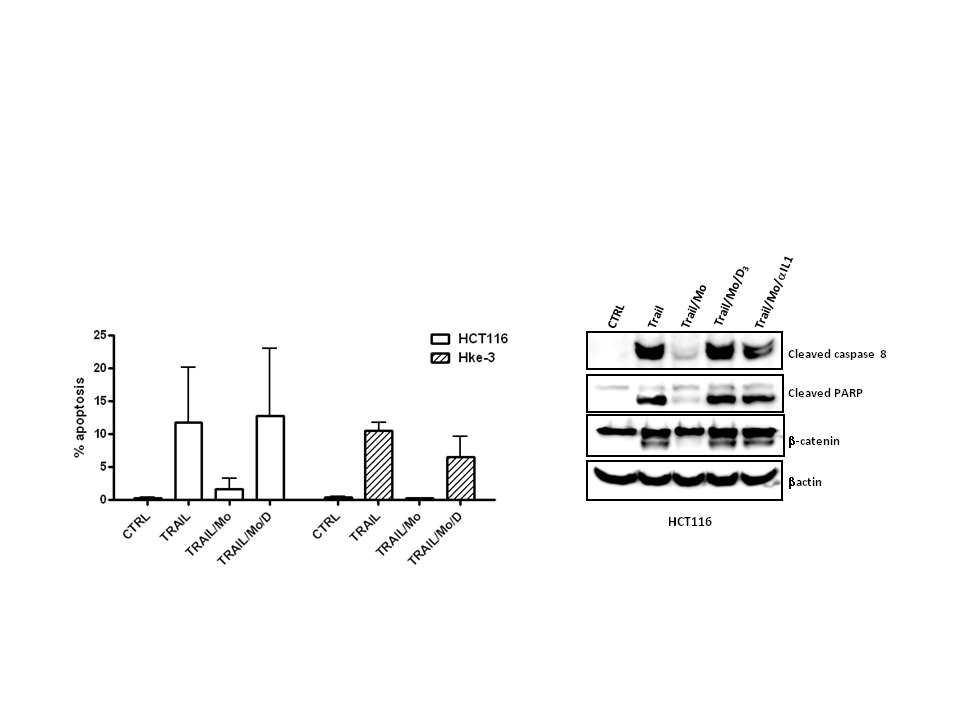

Supplement: Figure S2 — Vitamin D inhibits the anti-apoptotic activity of peripheral blood monocytes (Mo). HCT116 and Hke-3 cells were treated with TRAIL in the absence or the presence of peripheral blood monocytes and vitamin D, as indicated. The extent of apoptosis was determined by PI staining (left panel), and the activation of caspase 8 and cleavage of PARP and beta catenin were determined by immunoblotting (right panel). (0.12 MB TIF) [file pone.0011700.s002.tif]

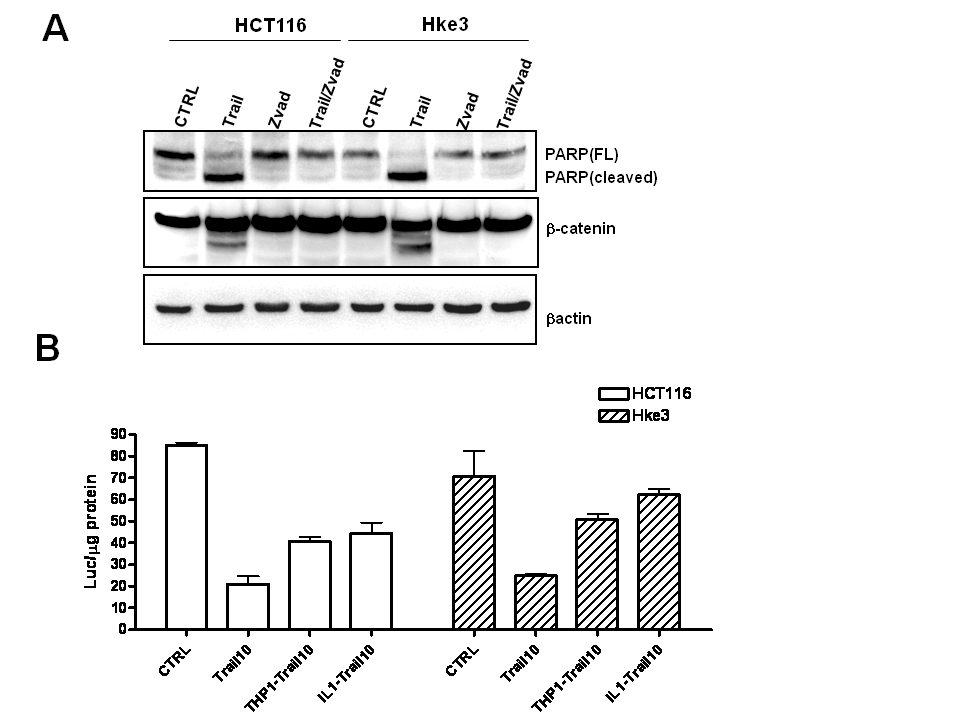

Supplement: Figure S3 — TRAIL inhibits beta-catenin/TCF4 transcriptional activity. A: HCT116 and Hke-3 cells were treated with TRAIL in the presence of pan-caspase inhibitor, ZVAD, and the cleavage of PARP and beta-catenin was determined by immunoblotting. B: HCT116 and Hke-3 cells were transfected with the TOP-FLASH reporter gene and were treated with TRAIL (10 ng/ml) in the presence of macrophages or IL1 as indicated for 24 hours. (0.16 MB TIF) [file pone.0011700.s003.tif]

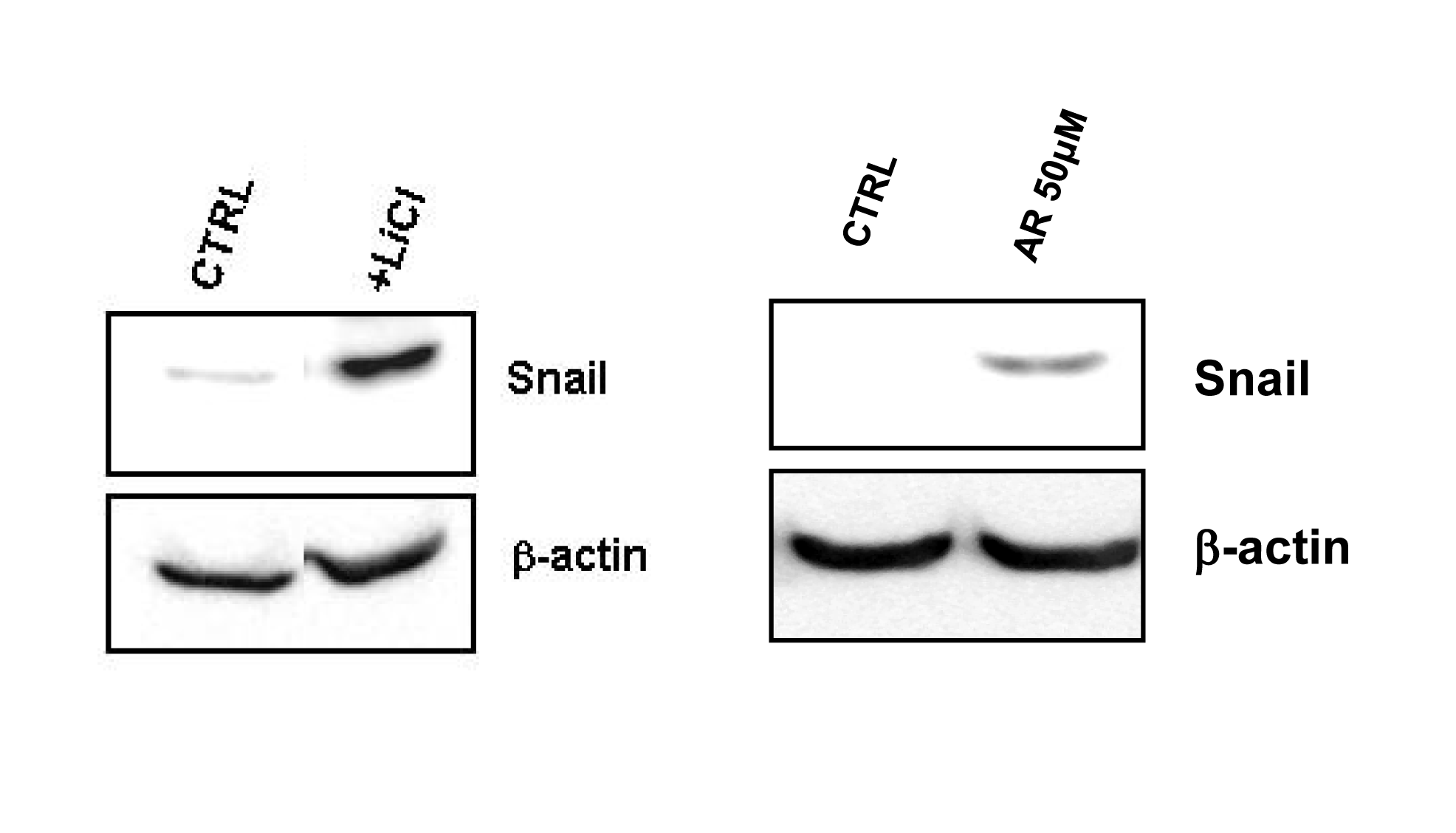

Supplement: Figure S4 — Inhibition of GSK3beta stabilizes Snail in tumor cells. HCT116 cells were treated with LiCl (10 mM) or with AR-A014418 (AR, 50 mM) for 24 hours and the levels of Snail and beta actin were determined by immunoblotting. (2.24 MB TIF) [file pone.0011700.s004.tif]
